# Acute respiratory illness among a prospective cohort of pediatric patients using emergency medical services in India: Demographic and prehospital clinical predictors of mortality

**DOI:** 10.1371/journal.pone.0230911

**Published:** 2020-04-02

**Authors:** Corey B. Bills, Jennifer A. Newberry, G. V. Ramana Rao, Loretta W. Matheson, Srinivasa Rao, Swaminatha V. Mahadevan, Matthew C. Strehlow

**Affiliations:** 1 Department of Emergency Medicine, University of Colorado, Aurora, Colorado, United States of America; 2 Department of Emergency Medicine, Stanford University, Palo Alto, California, United States of America; 3 GVK Emergency Management and Research Institute, Devar Yamzal, Telangana, India; Monash University School of Public Health and Preventive Medicine, AUSTRALIA

## Abstract

**Background:**

In India, acute respiratory illnesses, including pneumonia, are the leading cause of early childhood death. Emergency medical services are a critical component of India’s public health infrastructure; however, literature on the prehospital care of pediatric patients in low- and middle-income countries is minimal. The aim of this study is to describe the demographic and clinical characteristics associated with 30-day mortality among a cohort of pediatric patients transported via ambulance in India with an acute respiratory complaint.

**Methods:**

Pediatric patients less than 18 years of age using ambulance services in one of seven states in India, with a chief complaint of “shortness of breath”, or a “fever” with associated “difficulty breathing” or “cough”, were enrolled prospectively. Patients were excluded if evidence of choking, trauma or fire-related injury, patient was absent on ambulance arrival, or refused transport. Primary exposures included demographic, environmental, and clinical indicators, including hypoxemia and respiratory distress. The primary outcome was 7 and 30-day mortality. Multivariable logistic regression, stratified by transport type, was constructed to estimate associations between demographic and clinical predictors of mortality.

**Results:**

A total of 1443 patients were enrolled during the study period: 981 (68.5%) were transported from the field, and 452 (31.5%) were interfacility transports. Thirty-day response was 83.4% (N = 1222). The median age of all patients was 2 years (IQR: 0.17–10); 93.9% (N = 1347) of patients lived on family incomes below the poverty level; and 54.1% (N = 706) were male. Cumulative mortality at 2, 7, and 30-days was 5.2%, 7.1%, and 7.7%, respectively; with 94 deaths by 30 days. Thirty-day mortality was greatest among those 0–28 days (N = 38,17%); under-5 mortality was 9.8%. In multivariable modeling prehospital oxygen saturation <95% (OR: 3.18 CI: 1.77–5.71) and respiratory distress (OR: 3.72 CI: 2.17–6.36) were the strongest predictors of mortality at 30 days.

**Conclusions:**

This is the first study to detail prehospital predictors of death among pediatric patients with shortness of breath in LMICs. The risk of death is particularly high among neonates and those with documented mild hypoxemia, or respiratory distress. Early recognition of critically ill children, targeted prehospital interventions, and diversion to higher level of care may help to mitigate the mortality burden in this population.

## Introduction

Acute respiratory illnesses (ARIs) are a major cause of global morbidity and mortality, accounting for nearly one in five global childhood deaths.[1,2] Including pneumonia, ARIs kill more children than any other illness—more than AIDS, malaria and measles combined.[3] Globally, over 150 million new episodes of childhood pneumonia occur each year, with more than 95% of these cases occurring in developing countries.[4,5]

Over the last 30 years, the implementation of safe, effective and affordable interventions has reduced global mortality from pneumonia from 4 million in 1981[6] to approximately one million in 2013.[5] The Integrated Global Action Plan for Pneumonia and Diarrhoea has highlighted a number of interventions deemed crucial to reducing pneumonia mortality in children including adequate nutrition, reducing air pollution, vaccination, and access to appropriate treatment.[7] New management protocols have also helped providers in the early recognition and referral of children with severe disease to higher levels of care. However, protocols do not routinely comment on care during transport, the mode of transport, nor detail how to integrate referral processes across larger health systems.[8]

The need for improved care and referral processes for children with pneumonia is particularly critical in India, which accounts for the highest burden of new cases of pneumonia globally with an estimated incidence of 43 million in 2010.[9] In 2013, its largest burden of under-five mortality was attributable to pneumonia, with a mortality rate of 6.3 deaths per 1000 live births.[10] Fortunately, the opportunity for early intervention through prehospital care and timely interfacility transport exists in India where they are pioneering emergency medical services (EMS) development in low- and middle-income countries (LMICs).

In India, focus on increasing access to care and emergency referral to appropriate treatment has led to the massive growth of ambulance-based EMS. There was no dedicated centralized ambulance system until 2005. Since then, EMS has expanded to include a system based on a public-private partnership, currently with more than 17,000 emergency medical technicians (EMTs) providing care across 15 states and union territories.

Recent data suggests EMS is a critical component of the public health infrastructure and not only gets patients to care faster, but also to the appropriate level of care commensurate with acuity.[11,12] However, significant gaps remain in our understanding of the care of pediatric patients in the prehospital setting. First, while structured referral and transfer protocols for sick children in India exist, it is unclear what role ambulance-based transport has in the overall care of patients.[13] Second, there is limited to no available data on the specific care provided during transport, and none specific to respiratory illnesses.

This study helps to fill these gaps by describing the demographic, social, and prehospital clinical characteristics associated with 30-day mortality among a cohort of pediatric patients transported via EMS in seven states in India with acute shortness of breath.

## Methods

From June 20 to September 2, 2016, we enrolled a convenience sample of pediatric patients, less than 18 years old, who called a single EMS agency for a primary chief complaint of “shortness of breath”; or a primary chief complaint of “fever” with presence of “difficulty breathing” or “cough”. All patients who called with the aforementioned chief complaint, were enrolled Monday through Saturday during daytime hours (8 am-4 pm) across seven Indian states—Andhra Pradesh, Assam, Gujarat, Himachal Pradesh, Karnataka, Meghalaya and Telangana. In each of the respective Indian States, GVK EMRI operates as the sole centralized, public ambulance provider. Enrollment was during this time frame due to research assistant availability, safety and cost.

### Setting

At the time of this study GVK Emergency Management and Research Institute (GVK EMRI) operated across 15 states and union territories in India. Organizationally, GVK EMRI is a public-private enterprise, centralized at the state level. It is the largest single provider of ambulance-based services in the world operating over 7300 ambulances and approximately 130,000 calls per day.[14]

The majority of transports in this study are staffed by a driver and a single EMT. EMTs are trained in pediatric and neonatal resuscitation, including basic airway management, provision of supplemental oxygen, and administration of intravenous fluids and limited medications. Following initial assessment and treatment, ambulances transport patients to the nearest health facility, unless otherwise requested by the patient or family, or suggested by the referring healthcare provider in the setting of interfacility transfers (IFTs). Public health facilities include primary health centers (PHCs), community health centers (CHCs), district hospitals, and medical colleges, with neonatal and pediatric services increasing from basic (at PHCs) to more advanced (at district hospitals and medical colleges), however specific services vary from state to state. During transport EMTs may request medical guidance and direction for critical cases by phoning the call center to discuss with a physician. Reasons for calling include: to discuss treatment questions specific to protocol or to change the transport/referral plan, such as transport to a higher level of care.

### Study design

Any patient or individual (family member or healthcare provider) calling on behalf of a patient less than 18 years of age, for the aforementioned complaints were eligible for enrollment. Patients were included whether the transport originated in the field or at a health facility. Patients were excluded if reported evidence of choking, trauma or fire-related injury; patient was absent on ambulance arrival; or patients or their surrogate refused transport. Patient enrollment occurred at the time of initial EMT interaction using a standardized questionnaire to collect data in real-time by phone from the specific EMT caring for the patient. To not interfere with direct patient care, research assistants called the EMT after patient transport to gather additional information. At this time a patient’s guardian or immediate surrogate provided informed verbal consent for medical care, transport and follow-up per standard GVK EMRI ethics protocol. Verbal consent was witnessed directly by the EMT and then relayed to the research assistant.

EMTs collected data on demographics, clinical history, patient assessment, and ambulance-based interventions. EMTs also recorded two phone numbers at the time of patient enrollment with the aim of limiting loss to follow-up. Research assistants followed up with patient surrogates by phone at 2, 7, and 30 days. Follow-up data collection included patient status, hospital characteristics and admission, disposition, healthcare associated interventions, and follow-up care post-hospital discharge. Mortality was measured at 2, 7, and 30 days.

### Data analysis

Baseline demographic and prehospital care characteristics are provided as numbers and percents. Abnormal vital signs are by age, based on previously published criteria.[15] Hypoxemia was defined as a peripheral oxygen saturation (SpO_2_) less than 95% (mild), 93% (moderate), and 90% (severe). Presence of mild, moderate or severe respiratory distress was also assessed and based on EMT global assessment of the patient’s respiratory status. Additional clinical findings assessed by EMTs included respiratory rate, presence of accessory muscle use, subcostal or intercostal retractions, tracheal tugging, and presence or abnormality in lung sounds. Both height and weight were assessed and recorded on drop off at the health facility. Undernutrition was defined and categorized by WHO child growth standards and z scores, with underweight calculated as low weight-for-age; stunting as low height for age; and wasting as low weight-for-height.[16] A composite variable, malnutrition, was defined as presence of any of the three.

Time and distance to health facility was recorded in minutes and kilometers, respectively. Health facility type was categorized by funding type, whether government or private and by the level of care provided. Primary and community health facilities were categorized as low (i.e. lower care level) facilities, while medical colleges, district, private and government-supported hospitals were subcategorized as high-level facilities.

Seven and 30-day mortality related to demographics, clinical variables, EMT care, and hospital-based interventions were compared using chi-square analysis for categorical variables (or Fisher’s exact test when appropriate), and Wilcoxon two-sample test for continuous variables.

Univariable logistic regression was used to explore the association of individual variables with 30-day mortality. We constructed three separate multivariable logistic regression models. The first combined all transports. Two additional models stratified by transport type were also run, owing to significant differences in patient characteristics, possible predictors, and confounders of mortality at 30 days. In all three models, predictor variables were chosen from univariate analysis if the p-value was <0.10; and per methodology recommended by Lederer, et al; we also included those variables considered to be known historical risk factors for early childhood death from respiratory illness, known surrogates of critical illness in the prehospital period, and possible confounders.[17] The number of variables used in the final models differed between combined and stratified owing to the different number of absolute deaths in each. In each model we report the total number of patients in the model, the calculated area under the curve (AUC) to assess for discrimination, Hosmer-Lemeshow (H-L) statistics for goodness of fit, and variance inflation factor (VIF) statistics to assess for multi-collinearity. We report unadjusted and adjusted odds ratios (aORs) and 95% confidence intervals (CI) for predictor variables to the outcome variable: 30-day mortality. A p-value of <0.05 was considered statistically significant.

Institutional Review Boards at both Stanford (IRB#36066) and at GVK EMRI reviewed and approved this study. All data was securely collected and managed via REDCap (Stanford University), and analysis was conducted via SAS Enterprise Guide for Windows, V.4.3 (SAS Institute, Cary, North Carolina, USA).[18]

## Results

**Fig 1:**
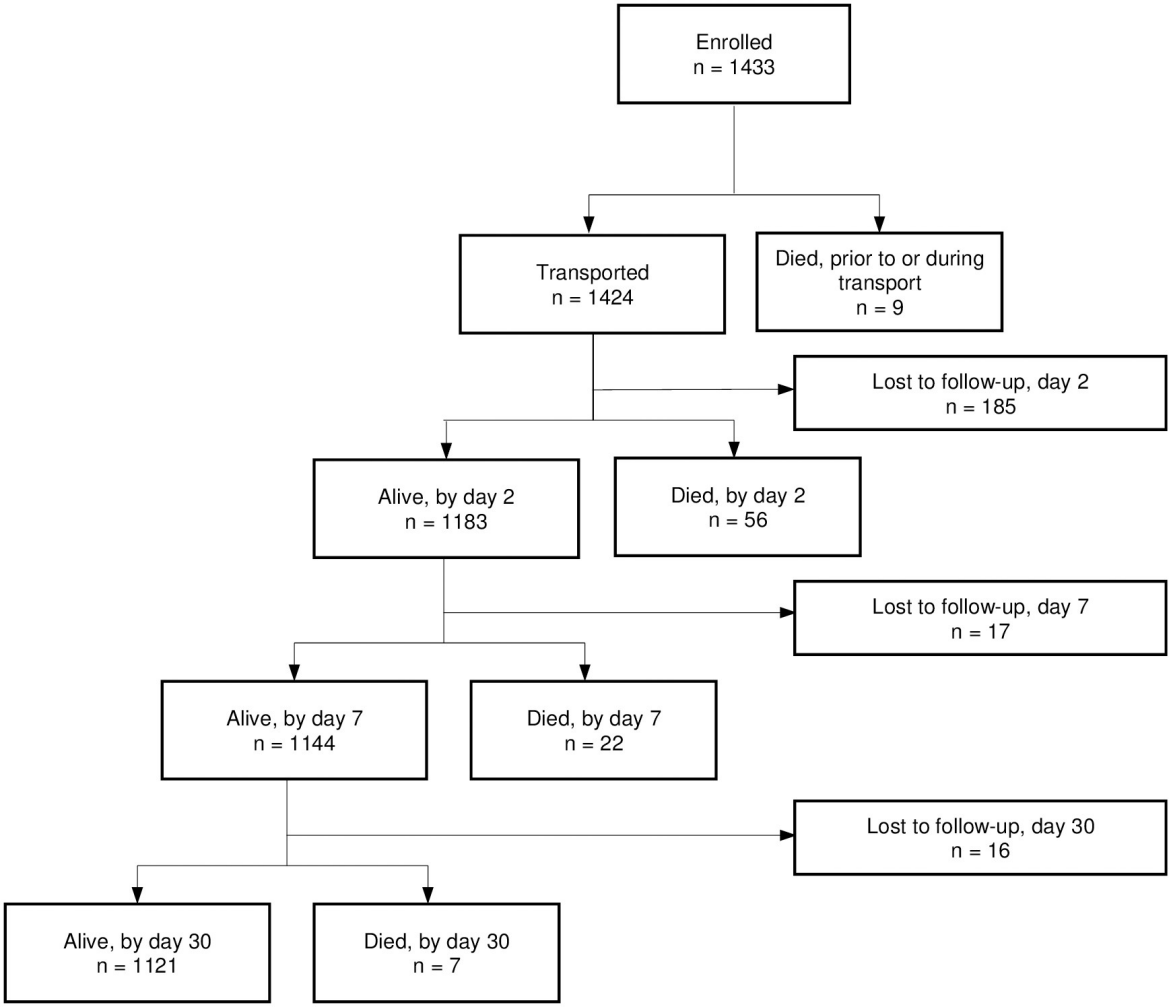
Flow diagram of patient study cohort.

A total of 1433 patients were enrolled during the nearly 11-week study period. (Fig 1) The median age of patients was 2 years (IQR: 0.16–10 years). (Table 1) A majority, 93.9% (n = 1346), were from families with incomes below the poverty level (as defined by family allocation of a government ration card to receive civil supplemental supplies such as rice and grains), and 68.5% (n = 982) were of a lower social caste as defined by national demographic categories. Approximately 78.2% (n = 1121) of calls occurred in rural or tribal areas.

**Table 1:** Demographic characteristics among a cohort of pediatric patients using EMS in seven states in India, June–September 2016.

Characteristic*	N = 1433	%
Age categories		
Median (IQR)**	2	2 m-10 y
0–28 days	285	20
29–364 days	276	19.3
1 year- 4 years	326	22.8
5 years-17 years	546	38.1
Sex		
Female	656	45.8
Male	776	54.2
State		
Andhra Pradesh	127	8.9
Assam	149	10.4
Himachal Pradesh	474	33.1
Gujarat	226	15.8
Karnataka	315	22
Meghalaya	51	3.6
Telangana	91	6.4
Geographic location		
Urban	308	21.5
Rural	993	69.3
Tribal	129	9.0
Social status***		
Backward caste	369	25.8
Other caste	427	29.8
Scheduled caste	345	24.1
Scheduled tribe	268	18.7
Economic Status****		
Below poverty level	1346	93.9
Above poverty level	83	5.8

*Missing data: sex (N = 1), geographic location (N = 4), social status (N = 24), economic status (N = 4).

**IQR: Interquartile range

*** Self-identified caste is used as a proxy for social status in India and is often used in national population health level monitoring. Scheduled caste is the lowest, most socially disadvantaged group. ‘Scheduled tribe’ is also a disadvantaged group. ‘Backward caste’ is an intermediary group socially; and ‘other caste’ includes all those who do not belong to the aforementioned groups and have the highest social status.

**** Economic status is defined by whether patients were dependent on the low-income government subsidy program (colored ration card).

In total, 981 (68.5%) patients were transported from the field. An additional 452 (31.5%) were IFTs. (Table 2) Over 98% of patients had a measured pulse, respiratory rate and documented lung exam. Slightly less patients had a measured temperature (83.5%) and SpO_2_ (81.9%). Clinical exam findings among IFTs were universally more abnormal (except temperature) as compared to field transports.

**Table 2:** EMT assessment, abnormalities, and interventions stratified by field transports and IFTs, among pediatric patients in seven states in India.

	Field		IFT	
	N = 981	%	N = 452	%
**EMT assessments**				
Mental status*				
Normal	927	94.5	386	85.4
Abnormal	41	4.2	66	14.6
Not measured	13	1.3	0	0
Heart rate				
Normal	654	66.7	333	73.7
Abnormal	308	31.4	117	25.9
Not measured	19	1.9	2	0.4
Respiratory Rate				
Normal	312	28.2	211	46.7
Abnormal	654	66.7	239	52.9
Not measured	15	1.5	2	0.4
Temperature				
Normal	481	49.0	258	57.1
Abnormal	366	43.2	92	26.3
Not measured	134	13.7	102	22.6
Blood pressure				
Normal	163	16.7	39	8.6
Abnormal	43	4.4	17	3.7
Not measured	775	79.0	396	87.6
Oxygen saturation**				
Normal	583	59.4	210	46.5
Abnormal	195	25.1	185	46.8
Not measured	203	20.7	57	12.6
Weight (kg) measured***	910	93.3	397	87.8
Height (cm) measured***	791	81.1	366	81.0
Nutrition****				
Normal	446	45.5	169	37.4
Undernutrition	250	25.5	139	30.8
Wasting	97	9.9	113	25.0
Stunting	266	27.1	91	20.1

* Mental status is by AVPU: normal is Alert; abnormal is responsive to Verbal, Painful and Unresponsive

**Abnormal oxygen is <95%

***kg: kilogram; cm: centimeter

**** Underweight: low weight-for-age; stunting: low height for age; and wasting: low weight-for-height.

EMTs gave supplemental oxygen to 768 (53.8%) patients. Of 825 patients with a documented abnormal respiratory rate, oxygen saturation <95%, or exam concerning for respiratory distress, 558 (71.3%) received supplemental oxygen. EMTs placed IVs in 100 (7.0%) patients, and 72 (5.1%) patients received IV fluid. EMTs attempted to reach physician support in 49.7% (n = 709) of cases, but attempted contact during IFT transports (n = 183, 40.5%) less often as compared to field transports (n = 526, 54.0%, p-value <0.001).

The median total transport time was 72 minutes (IQR: 47’-105’). (Table 3) Transport times were modestly shorter for transport from the field (68’ IQR: 43’- 96’) as compared to IFTs (80’ IQR: 55’- 120’; p<0.0001). Among all transported patients the median distance travelled from dispatch to scene was 7 km (IQR: 2–15 km); and the median scene to hospital distance travelled was 17 km (IQR: 9–29 km). Both time and distance from the scene to health facility were significantly longer among IFTs, almost double that of field transports.

**Table 3:** Facility characteristics and care stratified by field transports and IFTs, among pediatric patients in seven states in India.

Characteristic	Field		IFT		p-value
	N	% (IQR)	N	% (IQR)	
**Destination facility type**	978		450		
Government affiliated					
Public (government affiliated)	855	87.4	365	81.1	0.002
Private	123	12.6	85	18.9	
Facility level*					
Lower level	466	47.6	53	11.8	<0.001
Higher level	512	52.3	397	88.2	
Time from dispatch to facility (mins, IQR)**	64	(41–92)	77	(52–116)	<0.001
Distance from scene to facility (km, IQR)***	14	(7–23)	28	(15–43)	<0.001
Facility based care****					
Inpatient length of stay (days, IQR)	1	(0–4)	4	(2–7)	<0.001
At least 1 night in the ICU	125	12.8	162	36.1	<0.001
Endotracheal intubation	11	1.4	21	6.3	<0.001
Follow-up care scheduled	269	33.0	137	44.3	<0.001

*Lower level facilities include primary and community health centers; while high level facilities include medical colleges, district, private and government supported hospitals.

**Transport missing data: Time from dispatch (N = 27), distance from dispatch (N = 3); mins: minutes

***Facility missing data: Length of stay and ICU (N = 6), endotracheal intubation (284); km: kilometer

****Among those surviving to hospital discharge (N = 1124)

The facility category to which patients were transported also differed between IFTs and field transports. Among field patients 466 (47.6%) were taken to facilities with lower level of capacity, and 512 (52.3%) were taken to facilities that provide higher level of services. Conversely, a much higher proportion of IFTs were transferred to higher level facilities (N = 397, 88.2%; p-value <0.001). In most cases IFTs were transferred to a higher level of care after only a short duration (<12 hours) at the original hospital; N = 404, 89.4%.

Health facility-based care was collected via patients and families on 1202 patients. The majority reported receiving antibiotics in hospital (n = 850, 70.7%) and/or on discharge (n = 880, 77.2%). In total, 1215 patients either died or were followed to 30 days. Response rates were 87.1% at 2 days, 85.9% at 7 days, and 84.8% at 30 days. Overall cumulative mortality at 2, 7, and 30-days was 5.2%, 7.1%, and 7.7%, respectively; with a total of 94 deaths by 30-days. Stratified by age, the largest burden of mortality fell to those between 0–28 days with a mortality of 169.6 per 1000 patients at 30-days. Under-5 mortality (U5M) rate was 97.7 per 1000 patients at 30-days. IFT patients carried an almost three-fold risk of death as compared to scene transports (14% vs 5%, p<0.001).

Similarly, demographic characteristics associated with mortality at 30 days differed by whether the call originated from the field or was an IFT. (Table 4) Among field transports those that died were more likely to be less than a year old (p = 0.031), from an urban area (p = 0.001), of a lower social status (as designated by caste (p = 0.013), to be an only child (p<0.001), and to live in the home of a current smoker (p = .030). Comparatively, deaths among IFTs tended to live in a home with an open cook stove (p<0.001), a current smoker (p = 0.001) and with parents who were illiterate (p = 0.035).

**Table 4:** Patient demographic characteristics associated with 30-day mortality stratified by field transports and IFTs, in seven states in India, June–September 2016.

	Field				p-value*	IFTs				p-value*
	Alive		Dead			Alive		Dead		
	N = 812	%	N = 42	%		N = 309	%	N = 52	%	
Age					0.031					0.445
0–28 days	58	7.1	10	23.8		128	41.4	28	53.8	
29–364 days	137	16.9	12	28.6		61	19.7	9	17.3	
1–4 years	227	28.0	7	16.7		45	14.6	5	9.6	
>4 years	390	48.0	13	31.0		75	24.3	10	19.2	
Sex					0.714					0.149
Female	371	45.7	18	42.9		139	45.0	29	55.8	
Male	440	54.3	24	57.1		170	55.0	23	44.2	
Geography					0.001					0.204
Urban	92	11.4	12	28.6		114	36.9	24	46.2	
Rural/Tribal	716	88.6	30	71.4		195	63.1	28	53.8	
Social status					0.013					0.356
Other caste	279	35.3	7	16.7		90	29.3	12	23.1	
Non-Other caste	511	64.7	35	83.3		217	70.7	40	76.9	
Economic status					0.449					0.778
Below poverty	771	95.3	39	92.9		286	92.9	48	92.3	
Above poverty	38	4.7	3	7.1		22	7.1	4	7.7	
Parent literacy					0.154					0.035
Yes	684	84.4	28	75.7		240	77.9	30	63.8	
No	126	15.6	9	24.3		68	22.1	17	36.2	
Only child					<0.001					0.717
Yes	35	6.0	7	26.9		63	27.6	11	30.6	
No	553	94.0	19	73.1		165	72.4	25	69.4	
Smoker in home					0.030					0.001
Yes	130	16.3	11	29.7		102	33.1	27	57.4	
No	670	83.8	26	70.3		206	66.9	20	42.6	
Open cook stove					0.135					<0.001
Yes	197	24.3	13	35.1		76	24.6	24	51.1	
No	614	75.7	24	64.9		233	75.4	23	48.9	

*χ^2^ test

Among all transports, EMT-based assessment of mental status (OR 7.39 95% CI 4.40–12.43) and classification of moderate to severe respiratory distress were the strongest predictors of mortality (OR 6.80 95% CI 4.32–10.69) in univariable analysis. (Table 5)

**Table 5:** Unadjusted and adjusted odds of mortality at 30 days among all transported patients, and stratified by transport type, from multivariable regression modeling.

**Combined**	Unadjusted		Adjusted	
	OR	95% CI	OR	95% CI
Abnormal mental status	7.39	4.40–12.43	2.14	1.05–4.37**
Respiratory distress	6.80	4.32–10.69	2.67	1.48–4.84**
O2 saturation <93%	6.03	3.68–9.88	2.56	1.43–4.60**
Lower level facility	3.67	2.05–6.58	1.67	0.73–3.86
Age <28 days	3.41	2.19–5.30	1.87	1.02–3.45**
Interfacility transport	3.25	2.12–4.99	1.12	0.59–2.11
Urban area	2.74	1.76–4.27	1.84	1.02–3.35**
Open cook stove	2.44	1.55–3.84	2.40	1.36–4.24**
Lower social status	2.00	1.19–3.36	1.87	0.96–3.67
*N = 961, *AUC = 0*.*833*, *HL Chi2 3*.*28 (p-value 0*.*858)*, *VIF*: *1*.*22 (range*:*1*.*07–1*.*38)*				
** Statistically significant predictors in multivariable analysis (p<0.05).				
**Field**	Unadjusted		Adjusted	
	OR	95% CI	OR	95% CI
Abnormal mental status	3.23	1.07–9.73	1.14	0.21–6.18
Respiratory distress	3.65	1.77–7.53	2.00	0.78–5.09
O2 saturation <95%	3.68	1.69–8.01	2.41	1.01–5.73**
Age <28 days	4.06	1.90–8.67	3.52	1.36–9.14**
Urban area	3.11	1.54–6.29	3.06	1.29–7.24**
Lower social status	2.73	1.20–6.23	--	--
Lower level facility	2.58	1.27–5.26	--	--
Open cook stove	1.69	0.84–3.38	--	--
*N = 666, *AUC = 0*.*725*				
** Statistically significant predictors in multivariable analysis (p<0.05).				
**IFT**	Unadjusted		Adjusted	
	OR	95% CI	OR	95% CI
Abnormal mental status	6.63	3.44–12.78	2.43	1.03–5.71**
Respiratory distress	7.14	3.52–14.46	3.31	1.44–7.62**
O2 saturation <95%	5.91	2.74–12.73	3.82	1.50–9.72**
Open cook stove	3.20	1.71–5.99	3.82	1.76–8.33**
Lower level facility	3.11	1.66–5.82	2.98	1.28–6.95**
Age <28 days	1.65	0.91–2.98	--	--
Urban area	1.47	0.81–2.65	--	--
Social status	1.38	0.69–2.76	--	--
*N = 309, *AUC = 0*.*865*				
** Statistically significant predictors in multivariable analysis (p<0.05).				

At the univariable level, the subjective evaluation of respiratory distress was stronger than any one component of the clinical assessment of respiratory distress, including the presence of severe abnormal respiratory rate for age (OR 2.29 95% CI 1.00–5.27), accessory muscle use (OR 4.20 95% CI 2.67–6.62), retractions (OR 3.04 95% CI 1.66–5.58), or the combined presence of any of these three (OR 5.38 95% CI 3.42–8.48).

In multivariable analysis, prehospital presence of respiratory distress was the strongest predictor of 30-day mortality among all patients (aOR 2.67, 95% CI 1.48–4.84). Among patients followed to 30 days, the odds of dying were over two and a half times among those with a prehospital SpO_2_ <95% as compared to those with normal SpO_2_ (95% CI 1.43–4.60).

Field and IFTs had differing mortality predictors. Among field transports mortality was over four times as likely among neonates (aOR 4.06, 95% CI 1.90–8.67) as compared to those greater than 28 days. Among IFTs, mortality in those with respiratory distress were over three times the odds for mortality among those with normal breathing (aOR 3.31, 95% CI 1.44–7.62). Neither neonatal age nor urban geography was significantly associated with mortality in unadjusted or adjusted modeling among IFTs.

Based on the H-L statistic there is no evidence to suggest a lack of fit in any of the three models (Combined *χ*^2^ = 3.28, p-value = 0.858; Field *χ*^2^ = 2.00, p-value 0.367; IFT *χ*^2^ = 4.51, p-value 0.478). Additionally, there is minimal collinearity, with all models having a mean VIF <1.25 (Combined: mean VIF = 1.22, range: 1.07–1.38; Field mean VIF = 1.08 Range: 1.03–1.11; IFT mean VIF = 1.14, range: 1.02–1.22).

## Discussion

This is the first study to detail the prehospital demographics and clinical predictors of mortality among pediatric patients with shortness of breath in an LMIC. While ambulance services in India expeditiously connected critically ill children to hospital-based care, mortality among the young remained high. The risk of death was particularly elevated in neonates, patients with altered mental status, with respiratory distress or mild hypoxemia. Though characteristics associated with death were dissimilar between field and IFTs, presence of hypoxemia remained consistent.

Despite substantial progress in averting pediatric deaths from respiratory illnesses, such as pneumonia, high mortality continues to persist. In India, nearly half of all under-five deaths occur in the neonatal period (between 0–28 days).[19] Among a large meta-analysis of over 12000 pediatric patients with acute respiratory illnesses, the risk of death in those less than 2 months of age was 5-fold higher than older children.[20] Younger age was also significantly related to a higher risk of death in this study, with the highest mortality rate occurring among those less than 28 days old. Thirty-day under-five mortality was also 3–5 times that of published Indian national rates of death for acute respiratory illnesses.[21]

Likely this cohort represents a more critically ill segment of the population with most patients in this study spending at least one night in the hospital and subsequently provided hospital-based care. Alternatively, most national estimates of mortality are derived from all episodes not just those requiring hospitalization or care beyond that provided by a primary health center. This population represents an opportunity for intervention. Engaging both community members and hospital care providers to engage with ambulance services earlier in a patient’s illness has the potential to decrease delays to definitive care.

Several prehospital clinical variables, including respiratory distress and abnormal SpO_2_, were associated with 30-day mortality. Previous WHO Integrated Management of Childhood Illnesses (IMCI) guidelines largely ignored global assessments of respiratory distress and the importance of SpO_2_, instead focusing on indrawing.[22] Previous literature also suggested an inherent difficulty among healthcare providers in reliably assessing respiratory distress in low resource settings.[23,24] However in this study, EMT-based assessments were the greatest predictors of 30-day mortality.

Severe respiratory distress is also known to be a key predictor of decompensation and hypoxemia.[25] Similarly, in this study patients who had presence of both respiratory distress and SpO_2_ <95% the odds of mortality was nearly 10 times that of other patients (OR 9.92, 95% CI 5.96–16.51). Hypoxemia alone is a known indicator of poor outcomes.[26–29] Pooled estimates from 12 studies on nearly 14,000 children noted significant increased odds of death from acute respiratory illnesses (OR 5.47, 95% CI: 3.93–7.63) when hypoxemia was present.[30] In this study patients with an SpO_2_ <90% were more than six times as likely to die as compared to those with an SpO_2_ equal to or greater than 90% (OR 6.31, 95% CI 3.70–10.77). Oxygen saturation set at a more modest cutoff of <95% also had increased odds of death (OR 5.93, 95% CI 3.52–9.98). Previous data supporting a specific threshold for initiating supplemental oxygen is lacking, though most suggest an SpO_2_ of <90%, or 92% for children. The results of this study suggest that in the prehospital setting more liberal use of supplemental oxygen even for those with an SpO_2_ between 92–94% may be warranted.

Traditionally the costs of pulse oximetry were thought to be unaffordable, but recent evidence may suggest otherwise. Several pulse oximeters have been shown to be cost-effective[31], accurate[32], and validated in low resource settings.[33] This is the first study to comment on its use in prehospital systems in a LMIC. Given the relationship between hypoxemia and mortality, pulse oximetry could be incorporated into health system strengthening efforts in LMICs. Specifically, pulse oximetry should be included into protocols for prehospital triage and care—developing diversion systems for sick children to bypass lower level facilities and proceed directly to higher levels of care—and in optimizing interfacility referrals. Ensuring facility and ambulance-based providers are measuring oxygen saturation consistently is an important quality of care metric.

National recommendations and IMCI protocols are available throughout India to aid in the decision to transfer children to a higher level of care, but data specific to their use are lacking.[13,34] Furthermore, access to and capacity management at pediatric specialty centers is not well coordinated and ambulance diversion protocols to these centers in India is not always clear. In this study the majority of IFTs were to district hospitals and medical colleges, however 12% were transferred to PHCs and CHCs, lower level facilities.

Across several indicators, including mortality, IFTs represented a more ill cohort, as compared to field transports. Even in countries with mature systems, patients are at increased risk of morbidity and mortality during interfacility transport.[35] The risk of adverse outcomes can be mitigated however, with utilization of transfer protocols and guidelines to aid in destination decisions.[36–38] While patients are often triaged at the facility level, as in IFTs in this study, several issues are known to complicate the referral process in LMICs. First, decisions on destination hospital remain variable, often dependent on individual providers’ concerns or family wishes, rather than on formal protocols of care. Second, even after the decision to transfer is made, there is almost no communication with the prehospital providers nor receiving facility, which limits real-time resource informed decision making.[39]

Protocols for IFT ought to be regionally specific and synergistic with community, prehospital and healthcare facility integration. Targeted interventions, including protocols for care and referral, enhanced communication between prehospital and health facilities during and after transport, and improved documentation, have been shown to help strengthen health systems in LMICs[40] and may help to mitigate the high rates of death seen in this population.

The strengths of this study are limited by the sampling method. Given the large number of calls to the systems, as well as limited availability of research staff secondary to safety concerns of working at night we were unable to randomize enrollment. As a result we have no data on transports that occurred overnight or on Sundays. Based on prior epidemiologic data from the US we would expect mortality to be higher at night and on the weekends, however to date there is no data from India looking at diurnal variation of ambulance-based care. Second, the enrollment period was relatively short and may not have captured specific periods of high respiratory illness, such as influenza season.

In this study we have collected data on diagnoses, follow-up planning, and hospital-based care. Hospital records in India are of dramatically variable quality and there is limited accessibility of records between facilities. Therefore, follow up data was per family and surrogate report and we are unable to verify information via the medical record. Future studies aimed at linking prehospital and hospital health system records would augment understanding of the overall quality of care.

Lastly, the variation in care seeking behavior in India, and in other LMICs more broadly, limits generalization of the results of this study to other populations. We know very little about the differences in illness severity stratified by method of arrival to the hospital. Future studies looking at other methods of accessing hospital care would further characterize gaps in overall access to quality pediatric care.

In conclusion, ambulance-based services in LMICs have the potential to aid in the continued triage of sick children, while reliably transporting and caring for children with a complaint of shortness of breath. Existing guidelines should incorporate known risk factors from this study including assessment of respiratory distress and hypoxemia, with particular focus on neonates and interfacility transports, to aid in the development of integrated regionally-specific protocols for patient transport and care.
